# Sociodemographic Characteristics and Digital Behaviors Associated with the Use of Fitness and Diet Apps Among Adolescents

**DOI:** 10.34172/jrhs.2024.154

**Published:** 2024-07-31

**Authors:** Tatjana Gazibara, Milica Cakic, Jelena Cakic, Anita Grgurevic, Tatjana Pekemezovic

**Affiliations:** ^1^Institute of Epidemiology, Faculty of Medicine, University of Belgrade, Belgrade, Serbia

**Keywords:** Mobile applications, Physical fitness, Diet, Adolescent

## Abstract

**Background:** Numerous health apps focusing on fitness, nutrition, and physical activity are available, yet many adolescents have never used them. The purpose of this study was to assess the prevalence of the use of fitness and diet apps and related socio-demographic factors and digital behaviors among high school students.

**Study Design:** A cross-sectional study.

**Methods:** High school students were recruited from 4 out of 21 public high schools in Belgrade, Serbia. Participants filled out an anonymous questionnaire about socio-demographic characteristics and digital behaviors, along with the e-health literacy scale (eHEALS). Adjusted logistic regression was applied for data analysis using SPSS 20.

**Results:** Of the 702 students who completed the questionnaire, 670 used smartphones (95.4%; average age 16.5±1.2 years). The prevalence of fitness and diet app use among girls was 29.7% and 9.6%, as well as 17.3% and 3.6% among boys, respectively. Having higher family income, having better e-health literacy, browsing websites about fitness and diet, and using diet apps but not watching YouTube were associated with the use of fitness apps among girls. Being younger and browsing fitness websites and YouTube were associated with the use of fitness apps among boys. Being younger, browsing websites about diet, and using fitness apps were associated with the use of diet apps among girls.

**Conclusion:** Based on the findings, girls used fitness and diet apps more often than boys. Practical demonstrations on how to utilize certain health apps could be an additional opportunity to support positive health behaviors among adolescents.

## Background

 Smartphones are increasingly used among adolescents not only to keep in contact with classmates and peers but also to browse the Internet and YouTube and access software applications (apps).^1^ A recent study on adolescents from Portugal has identified that features such as food and physical activity recommendations are critical for the use of a diet app to prevent overweight and obesity.^2^ This study has also found that the utility and interest in a particular health app were the main factors in favor of their continued use.^2^ Presently, although numerous health apps focusing on fitness, nutrition, and physical activity are available,^3^ many adolescents have never used them.^2^

 To help adolescents be more physically active and learn about healthy diet and nutrition, the use of technology and online digital platforms could be a valuable tool in efforts to improve adolescents’ health and health-related behaviors.^4^ Bearing in mind that positive health-related attitudes and behaviors established in adolescence set the trajectory for healthy lifestyles later in life, the examination of Internet use could offer important evidence about digital health information needs among young people.

 Accordingly, this study aimed to assess the prevalence of the use of fitness and diet apps and to examine contributing socio-demographic factors and digital behaviors among high school students.

## Methods

 A cross-sectional study was performed from December 2016 to January 2017. Four out of 21 public high schools were randomly selected from the inner urban area of Belgrade, the capital city of Serbia.^5^ In all schools, both girls and boys are enrolled together, so classrooms always include students of both genders. High schools have four-year programs and offer general education in the natural sciences, humanities, foreign languages, arts, and physical education. After the completion of high school, the majority of students continue their education at universities. For this study, the online sample size calculator was used,^6^ with a margin of error of 5%, a 99% confidence level, the suggested high school population size of 20 000 from the 2011 Census,^7^ and a population distribution of 50%. The calculated minimum sample size was 655, which was increased by approximately 10% because potential students refused to participate. All students who were invited also consented to participation, as high school students were quite interested in taking part in this study. Therefore, the response rate was 100%.

 Ethical approval for the study was obtained from the Institutional Review Board of the Faculty of Medicine, University of Belgrade (approval No. 747-I). As the study participants were minors, the schools informed the parents about the study. The parents were offered the possibility of opting out, implying that the parents could have informed the school in case they did not want their child to participate in the survey. Assent (i.e., consent for participation by participants who were minors) was implied by returning the completed questionnaire.

###  Observed outcome

 The students were asked to circle the apps that they used at the time of the survey for (1) fitness, running, counting of steps/distance, and the like, as well as (2) healthy diet, nutrition, meal preparation, calorie counting, and the like. The use of fitness and diet apps was categorized as a binary value (yes vs. no) based on whether the specific type of app was circled. A space was considered for students to write the name of an app that they utilized in case they were unsure how to classify it.

###  Covariates

 The students were asked about their socio-demographic characteristics (gender and age), study year (1^st^ to 4^th^), type of high school program (science-mathematics vs. humanities-languages), grade point average (GPA), parental education level, and household monthly income. In the Serbian school system, the GPA is based on a numerical scale from 1 to 5 (1 = fail, 2 = pass, 3 = good, 4 = very good, and 5 = excellent). Higher grades correspond to higher academic achievement. The possible range of GPA is 2.0 (poor) – 5.0 (excellent). Students are graded at the end of the winter and the spring semester. However, only grades at the end of the spring semester (which is the end of the academic year) are registered as the final grading score for that school year. Higher grades correspond to higher academic achievement. Students were asked to provide the highest education attainment for both parents. Because there were < 5 observations for the primary education category, primary and secondary education were merged into one. Household monthly income was categorized into < 405, 405–810, and > 810 Euros per month based on the average income in the capital city area.

 To examine the use of the Internet, the students were asked, “Do you use the Internet?” (yes vs. no). They were also asked to write about the age at which they first began using the Internet and specifically asked whether they browsed webpages about fitness/exercise and nutrition/diet (yes vs. no). In addition to webpages, students were asked whether they used YouTube as a source of health-related information (yes vs. no). To examine the use of smartphones, the students were questioned, “Do you use a smartphone?” (yes vs. no).

 E-health literacy, defined as the ability to search, comprehend, and assess online health information to make informed health decisions,^8^ was quantified using the e-health literacy scale (eHEALS).^9^ The scale is composed of 8 questions that examine students’ confidence when searching, assessing, and using health information on the Internet. Each question offers 5 answers (ranks), whereby rank 1 = strongly disagree and rank 5 = strongly agree. The sum of all 8 ranks represents the total eHEALS score (range 8–40). Better e-health literacy is quantified by a higher score. The eHEALS was previously translated and validated in the local language. The internal consistency, as measured by Cronbach’s alpha coefficient, was 0.849.^10^ One factorial structure was corroborated using the confirmatory factor analysis (goodness of fit index = 0.983, comparative fit index = 0.964, Tucker Lewis index = 0.949, root mean square error of approximation = 0.090).

 Finally, the students were asked to quantify the degree of influence of online health information on their health decisions. Their answers were graded on a 4-point scale (1 = not at all, 2 = a little, 3 = quite a bit, and 4 = a lot).

###  Data analysis

 Mann-Whitney (for continuous variables) and Chi-square (for categorical variables) tests were used to assess differences between variables. Considering that it has been consistently observed that females tend to practice more favorable health behaviors than males,^11^ the interaction terms (gender x age) in the two regression models (Supplementary file 1, Tables S1 and S2) were tested using the PROCESS macro by Andrew Hayes. The PROCESS macro is a software add-on for SPSS and SAS that can be downloaded from the open-source website http://processmacro.org. The purpose of the program is to facilitate the moderation and mediation analysis of variables in different regression models.

 Two series of multiple logistic regression models were built to examine factors associated with the use of fitness and diet apps. In the first one, the dependent variable was the ‘use of fitness apps’ (yes vs. no). In the second series, the dependent variable was the ‘use of diet apps’ (yes vs. no). The independent variables were classified according to Model 1 (with demographic variables only), Model 2 (demographic variables + digital consumer behaviors), and Model 3 (demographic variables + digital consumer behaviors + use of apps). All analyses were performed using Statistical Package for Social Sciences (SPSS Inc., Chicago, IL, USA), version 20, and *P* < 0.05 was considered statistically significant.

## Results

 A total of 702 high school students filled out the questionnaire. All students used the Internet. Of 702 students, 670 (95.4%) utilized smartphones and were able to access fitness and diet apps from their mobile phones, so only their data underwent analysis. Socio-demographic characteristics and digital behaviors of students who employed smartphones are presented in Table 1. There were significantly more students in the science-mathematics program compared to those in the humanities-languages program, and girls had a higher GPA than boys. Boys started using the Internet at an earlier age than girls. Girls used fitness and diet apps more than boys. Girls browsed fitness and diet webpages more than boys. No difference was observed in the use of YouTube between girls and boys (Table 1).

**Table 1:** Socio-demographic characteristics and digital behaviors of high school students who used smartphones

**Categorical variables**	**Girls, n=394**		**Boys, n=276**		***P*** ** value**
	**Number**	**Percent**	**Number**	**Percent**	
Type of high school program					
Science-mathematics	204	51.8	171	62.0	0.009
Humanities-languages	190	48.2	105	38.0	
Highest education attainment of the parents					
Primary or secondary	105	26.6	63	22.8	0.261
University	289	73.4	213	77.2	
Household monthly income (€)					
< 405	33	8.4	23	8.3	0.079
405-810	186	47.2	107	38.8	
> 810	175	44.4	146	52.9	
Influence of online information					
Not at all	81	20.6	57	20.7	0.070
A little	120	30.5	97	35.1	
Quite a bit	176	44.7	99	35.9	
A lot	17	4.4	23	8.3	
Use of fitness apps	117	29.7	51	18.5	0.001
Use of nutrition apps	38	9.6	10	3.6	0.003
Browsing fitness websites	229	58.1	126	45.7	0.001
Browsing websites about diet	183	46.4	60	21.7	0.001
Browsing YouTube	95	24.1	74	26.8	0.428
Continuous variables	**Mean**	**SD**	**Mean**	**SD**	***P*** **-value**
Age (year)	16.0	3.0	17.0	3.0	0.456
Grade point average	4.6	0.9	4.5	0.9	0.001
Age at first internet use in years	10.0	3.0	9.0	3.0	0.001
eHEALS score	26.2	9.0	25.4	11.0	0.112

*Note*. eHEALS: E-health literacy scale; SD: Standard deviation.

###  Use of fitness apps

 A total of 117 (117/394, 29.7%) girls and 51 (51/276, 17.3%) boys who had smartphones utilized fitness apps (χ^2^ = 10.870, *P* = 0.001) listed in the questionnaire. Overall, only three students used fitness apps that were labeled as “other” (i.e., not listed in the questionnaire). The multiplicative interaction of gender and age was tested to examine the role of gender in fitness app use. The product term of age and gender in the logistic regression model was deemed significant (Table S1). Accordingly, the study sample was stratified by gender. A total of three logistic regression models were tested in each stratum.

 The series of three regression models among the girls is presented in Table 2. Model 1 showed that girls whose families had higher monthly incomes were more likely to use fitness apps. Model 2 demonstrated that, in addition to higher monthly income, having better e-health literacy, browsing websites about fitness and diet, and a lack of use of YouTube were associated with the use of fitness apps. Model 3 indicated that, in addition to previous variables, the use of diet apps was associated with the use of fitness apps among girls.

**Table 2:** Factors associated with the use of fitness apps among high school girls

**Variables**	**Model 1**		**Model 2**		**Model 3**	
	**OR (95% CI)**	***P *** **value**	**OR (95% CI)**	***P *** **value**	**OR (95% CI)**	***P *** **value**
Age	0.97 (0.80, 1.17)	0.747	0.94 (0.76, 1.17)	0.569	1.01 (0.81, 1.27)	0.918
Type of school program (science vs. humanities)	1.21 (0.78, 1.89)	0.391	1.02 (0.62, 1.69)	0.923	1.03 (0.61, 1.73)	0.913
Grade point average	0.63 (0.40 1.01)	0.053	0.61 (0.36, 1.03)	0.062	0.58 (0.34, 1.00)	0.052
Parental education level (primary/secondary vs. university)	0.86 (0.51, 1.45)	0.566	0.57 (0.31, 1.06)	0.075	0.60 (0.32, 1.12)	0.112
Family income	1.81 (1.23, 2.67)	0.003	1.94 (1.25, 3.01)	0.003	1.75 (1.11, 2.76)	0.016
Age at first internet use	-	-	0.95 (0.85, 1.07)	0.442	0.96 (0.85, 1.08)	0.516
eHEALS score	-	-	1.11 (1.06, 1.16)	0.001	1.11 (1.06, 1.16)	0.001
Degree to which online information affect students’ health decisions	-	-	0.97 (0.70, 1.33)	0.843	0.98 (0.70, 1.36)	0.890
Browsing fitness websites	-	-	5.79 (3.24, 10.37)	0.001	5.26 (2.90, 9.57)	0.001
Browsing websites about diet	-	-	2.17 (1.28, 3.68)	0.004	1.80 (1.04, 3.11)	0.036
Browsing YouTube	-	-	0.47 (0.25, 0.88)	0.018	0.46 (0.24, 0.88)	0.020
Using diet apps	-	-	-	-	5.70 (2.44, 13.31)	0.001

*Note*. eHEALS: E-health literacy scale; OR: Odds ratio; CI: Confidence interval.

 The series of three regression models among the boys is provided in Table 3. Model 1 revealed that younger boys were more likely to use fitness apps. Model 2 represented that, in addition to younger age, browsing fitness websites and YouTube was associated with the use of fitness apps among boys. The results did not materially change after the adjustment for the use of diet apps (Model 3).

**Table 3:** Factors associated with the use of fitness apps among high school boys

**Variables**	**Model 1**		**Model 2**		**Model 3**	
	**OR (95% CI)**	***P *** **value**	**OR (95% CI)**	***P *** **value**	**OR (95% CI)**	***P *** **value**
Age	0.70 (0.52, 0.94)	**0.016**	0.75 (0.55, 1.02)	0.064	0.71 (0.52, 0.98)	0.036
Type of school program (science vs. humanities)	0.61 (0.35, 1.06)	0.078	0.64 (0.38, 1.13)	0.126	0.64 (0.36, 1.13)	0.124
Grade point average	0.76 (0.39, 1.46)	0.408	0.89 (0.44, 1.82)	0.753	0.80 (0.39, 1.67)	0.560
Parental education level (primary/secondary vs. university)	1.43 (0.66, 3.08)	0.368	1.51 (0.63, 3.63)	0.359	1.39 (0.57, 3.37)	0.467
Family income	0.98 (0.60, 1.60)	0.938	0.81 (0.47, 1.42)	0.470	0.83 (0.47, 1.45)	0.510
Age at first internet use	-	-	1.00 (0.88, 1.15)	0.965	1.02 (0.89, 1.17)	0.813
eHEALS score	-	-	1.03 (0.98, 1.09)	0.209	1.03 (0.98, 1.08)	0.215
Degree to which online information affect students’ health behavior	-	-	1.42 (0.96, 2.10)	0.082	1.43 (0.96, 2.13)	0.078
Browsing fitness websites	-	-	2.57 (1.24, 5.36)	0.012	2.51 (1.20, 5.23)	0.014
Browsing websites about diet	-	-	0.98 (0.43, 2.20)	0.955	0.96 (0.43, 2.17)	0.928
Browsing YouTube	-	-	3.67 (1.84, 7.33)	0.001	3.13 (1.53, 6.41)	0.002
Using diet apps	-	-	-	-	3.74 (0.82, 17.08)	0.089

*Note*. eHEALS: E-health literacy scale; OR: Odds ratio; CI: Confidence interval.

###  Use of diet apps

 A total of 38 (38/394, 9.6%) girls and 10 (10/276, 3.6%) boys who used smartphones also employed diet apps (χ^2^ = 8.848, *P*= 0.003). The interaction terms were tested to examine the role of gender as a moderator in the association between students’ age and the use of diet apps. The product term between age and gender was deemed significant (Table S2). For this reason, the study sample was stratified by gender. Because of the small proportion of boys who reported the use of diet apps (3.6%), the logistic regression models were not deemed sufficiently robust. Thus, factors associated with the use of diet apps were investigated only among the girls.

Table 4 lists the series of three regression models among the girls. Model 1 demonstrated that younger girls were more likely to use diet apps. Model 2 showed that being younger, having a higher family income, and browsing websites about diet and fitness were associated with the use of diet apps. After the adjustment for the use of fitness apps, Model 3 indicated that girls who were younger, browsed websites about diet, and used fitness apps were more likely to utilize diet apps.

**Table 4:** Factors associated with the use of diet apps among high school girls

**Variables**	**Model 1**		**Model 2**		**Model 3**	
	**OR (95% CI)**	***P *** **value**	**OR (95% CI)**	***P *** **value**	**OR (95% CI)**	***P *** **value**
Age	0.68 (0.50, 0.93)	**0.015**	0.65 (0.47, 0.91)	**0.012**	0.69 (0.49, 0.96)	**0.029**
Type of school program (science vs. humanities)	1.19 (0.54, 2.61)	0.666	1.28 (0.54, 3.01)	0.570	1.60 (0.66, 3.88)	0.300
Grade point average	1.15 (0.58, 2.29)	0.682	0.98 (0.47, 2.06)	0.970	0.98 (0.46, 2.13)	0.969
Parental education level (primary/secondary vs. university)	0.89 (0.39, 2.03)	0.780	0.65 (0.26, 1.60)	0.350	0.75 (0.29, 1.93)	0.557
Family income	2.32 (1.22, 4.42)	0.010	2.25 (1.14, 4.47)	0.020	1.80 (0.89, 3.68)	0.104
Age at first internet use	-	-	0.98 (0.83, 1.16)	0.853	0.99 (0.83, 1.18)	0.917
eHEALS score	-	-	1.04 (0.98, 1.11)	0.199	1.01 (0.94, 1.08)	0.877
Degree to which online information affect students’ health behavior	-	-	0.89 (0.55, 1.45)	0.650	0.94 (0.57, 1.54)	0.816
Browsing websites about diet	-	-	3.48 (1.51, 8.02)	0.003	2.60 (1.11, 6.09)	0.028
Browsing fitness websites	-	-	3.55 (1.44, 8.77)	0.006	2.13 (0.81, 5.61)	0.127
Browsing YouTube	-	-	0.87 (0.37, 2.01)	0.740	1.06 (0.43, 2.57)	0.903
Using fitness apps	-	-	-	-	5.51 (2.31, 13.18)	0.001

*Note*. eHEALS: E-health literacy scale; OR: Odds ratio; CI: Confidence interval.

## Discussion

 The results of this study revealed that high school girls used fitness apps and diet apps more than high school boys. Similar results were observed among adolescents in Flanders (Belgium), where fitness apps garner more interest than diet apps,^12^ as well as in the United Kingdom.^13^ However, in Vietnam, few teenagers use this type of app.^14^ This difference is expected, given that access to smartphones and, subsequently, health apps might be lower in resource-limited settings.

 The stratification of the study sample according to gender suggested that browsing fitness websites was associated with the use of fitness apps among high school girls and boys. The finding that high school girls used fitness and diet apps more frequently compared with high school boys is in line with a well-acknowledged difference between genders relative to self-care and health-promoting behavior.^15,16^ Females tend to practice more favorable health behaviors and are more interested in health compared to males.^16^ Women are also more inclined to seek advice or help.^15^ Thus, the results of our study support the previous evidence^15,16^ and indicate that the gender gap in digital health-information-seeking begins as early as adolescence.

 A previous study reported that the use of health apps helps set health goals and enable self-monitoring and self-perception.^17^ For an app to be used by adolescents, several researchers agree that the content needs to be easy to navigate, provide understandable information, and offer a personalized regimen, as well as the possibility to connect with peers and other people through social networks.^13,18,19^ Although health apps have the potential for health promotion among youth, not all health apps are equally effective in helping the users to achieve higher levels of fitness, desired body image/composition or ensure long-term health benefits. For example, one randomized controlled trial reported that, after a 2-month follow-up, the cardio-respiratory fitness of adolescents who used a specific fitness app did not differ from those who did not utilize such an app.^13^ Use of fitness and diet apps has also been observed to have a negative impact on some young adults because adjustment to the scheduled app program led them to feel socially isolated (due to a certain eating and fitness regimen), controlled by the app, or afraid of not being able to achieve certain targets.^2^

 Younger girls in our study were more likely to use diet apps. This finding could be explained by the notion that girls become aware of their body image at a younger age and strive to be thin compared to boys.^20^ Overall, it is estimated that one-half of adolescent girls are not satisfied with their weight and are more likely to diet and restrict caloric intake.^20^ Parental attitudes and behaviors toward food and dieting are closely related to those of their adolescent children.^21^ Furthermore, parental feedback about fitness and diet is the strongest contributor to body dissatisfaction among adolescents.^22^ Considering the risk of becoming underweight or developing eating disorders, the nutrition and diet of adolescent girls need to be closely monitored by adults (parents and school teachers). In fact, it is essential that parents be healthy role models for their adolescent children. Therefore, the parental role must be continuously emphasized in the effort to set the trajectory for adolescents’ healthy eating habits.

 In this study, it was found that girls coming from higher-income families were more likely to use fitness and diet apps. This was not observed in boys. Evidence suggests that there is an inverse association between adolescent girls’ weight and socio-economic status, with girls of higher socio-economic status more likely to be in the healthy weight range.^23^ A longitudinal study in the Czech Republic reported that adolescents from higher socio-economic groups were more likely to eat fruits and vegetables every day compared to those from lower socio-economic groups.^24^ Although socio-economic inequalities have a strong influence on adolescents’ food choices, the importance of in-person education about proper nutrition should be prioritized in schools using formal and informal teaching methods.

 Our findings revealed that the use of fitness and diet websites was consistently associated with the use of fitness and diet apps, respectively. This was identified among both girls and boys. Students in our sample began using the Internet at the age of 9 (boys) and 10 (girls), suggesting that adolescents in our study practically grew up using the Internet for various purposes. As a result, a high level of digital literacy is expected. For this reason, the association of better e-health literacy with the use of fitness apps among girls was not surprising. Given that adolescents’ use of the Internet in general preceded owning and using a personal smartphone, the use of various digital platforms, such as in our study (use of web pages, YouTube, and smartphone app) can be considered a ‘digital package deal’.

 Use of YouTube was associated with the use of fitness and diet apps among adolescent boys, but not among girls. Over the past years, the number of YouTube users has been growing. This digital platform offers unique audio-visual content that adolescents can relate to, and it has become a popular medium for sharing various health information, including data about fitness and diet. While YouTube content may be visually appealing for adolescent viewers, a recent study on YouTube videos aimed at achieving fitness reported that the content of fitness creators on YouTube was not health-promoting.^25^ Because of the visually appealing content, often documenting fitness transformations and ‘how to’ recommendations, it is reasonable to expect that the use of fitness and diet apps on YouTube is being endorsed. Considering the simultaneous use of various digital media, the importance of a balance between on-screen and off-screen time, parental involvement, and the improvement of digital literacy has been widely emphasized due to their relevance for the promotion of adolescents’ health.^26^

 The limitations of this study are related to the fact that high school students living in areas less urbanized than the capital city and rural areas have less access to the Internet, given that 63.8% of households have Internet access.^7^ We targeted high schools in the inner urban area, whereas high schools in municipalities on the outskirts of the metropolitan area were not included in our study. High school students in this region may have different behaviors and practices for smartphone and app use. In a similar vein, students from rural areas might have less access to smartphones due to costs. All fitness and nutrition-related apps were observed jointly. We have not stratified apps according to specific targets, such as weight loss/calorie counting, consistency of a certain diet, or exercise regimen. Nevertheless, because of the relatively low prevalence of diet app use, particularly among boys, further stratification according to types of fitness and diet apps would not allow a regression model to converge. We are aware that this response rate may be open to social acceptability bias, implying that students who were hesitant to participate did so because their classmates opted to participate. Due to the cross-sectional study design, we could not make definite causal inferences between the examined variables and the outcomes of interest. Finally, because of the fast pace of innovations in the digital landscape, the data presented in this study may not be entirely applicable to all school settings. However, despite the efforts to keep up, digital approaches to learning in schools in Serbia still face significant challenges.^27^ For this reason, our results could remain relevant in the forthcoming years.

**Highlights**

- The prevalence of app use was higher among high school girls than high school boys.
- Fitness apps were used more than diet apps.
- The use of YouTube was associated with fitness apps among boys.
- The use of Internet websites was related to fitness and diet apps.

## Conclusion

 In general, fitness and diet apps are being used among high school students. The prevalence of app use was higher among high school girls compared to high school boys. The use of fitness and diet websites was consistently associated with the use of fitness and diet apps across genders. Additionally, the use of YouTube was associated with the use of health apps among boys. Given that younger girls are more likely to be the users of diet apps, parents and other adults are advised to monitor girls’ diet regimens to prevent unhealthy dieting. Apps could be a useful tool to improve physical activity and nutrition. It is recommended that certain classes at school be devoted to the use of digital health resources. Practical demonstrations of the use of certain health apps could be an additional opportunity to support positive health behaviors among adolescents.

## Acknowledgements

 The authors are grateful to all high school students who participated in this study.

## Authors’ Contribution

**Conceptualization:** Tatjana Gazibara, Milica Cakic, Jelena Cakic, Anita Grgurevic, Tatjana Pekmezovic.

**Data curation:** Tatjana Gazibara, Milica Cakic, Jelena Cakic.

**Formal analysis:** Tatjana Gazibara.

**Funding acquisition: **Tatjana Pekmezovic.

**Investigation:** Tatjana Gazibara, Milica Cakic, Jelena Cakic, Anita Grgurevic, Tatjana Pekmezovic.

**Methodology:** Tatjana Gazibara, Milica Cakic, Jelena Cakic, Anita Grgurevic, Tatjana Pekmezovic.

**Project administration:** Tatjana Gazibara.

**Resources:** Tatjana Pekmezovic.

**Supervision:** Anita Grgurevic.

**Validation:** Tatjana Gazibara, Milica Cakic, Jelena Cakic, Anita Grgurevic, Tatjana Pekmezovic.

**Visualization:** Tatjana Gazibara.

**Writing–original draft:** Tatjana Gazibara.

**Writing–review & editing:** Milica Cakic, Jelena Cakic, Anita Grgurevic, Tatjana Pekmezovic.

## Competing Interests

 The authors declare that they have no conflict of interests.

## Ethical Approval

 The Institutional Review Board of the Faculty of Medicine at the University of Belgrade approved this study (approval No. 747/I).

## Funding

 The study was supported by the Ministry of Education, Sciences, and Technological Development of the Republic of Serbia (grant No. 200100).

## Supplementary Files

Supplementary file 1 contains Tables S1 and S2.
