# Supplementary material for: Sociodemographic Characteristics and Digital Behaviors Associated with the Use of Fitness and Diet Apps Among Adolescents
Source: J Res Health Sci. 2024 Jul 31;24(3):e00619. doi: 10.34172/jrhs.2024.154 (PMC11380736; doi:10.34172/jrhs.2024.154)
Supplement: Supplementary file 1 — contains Tables S1 and S2. [file jrhs-24-e00619-s001.pdf]

doi:10.34172/jrhs.2024.154
JRHS 2024; 24(3):e00619

Supplemental Table 1. Multiplicative interaction testing of age and gender: results of the multiple logistic regression model when the dependent variable was ‘use of fitness apps’

Model	B	SE	P-value	LLCI	ULCI
Constant	9.277	4.8764	0.0571	-0.2800	18.8352
Age	-0.6474	0.2853	0.0232	-1.2066	-0.0883
Gender	-4.6009	2.6664	0.0844	-9.8270	0.6251
Age x Gender	0.3201	0.1629	0.0494	0.0009	0.6394
School program	0.0719	0.1878	0.7017	-0.2961	0.4399
GPA	-0.3619	0.1784	0.0425	-0.7116	-0.0123
Fitness websites	1.3757	0.2033	0.0000	0.9771	1.7742
First internet use	-0.0201	0.0412	0.6255	-0.1010	0.0607

GPA-grade point average; B-unstandardized coefficient, se-standard error, p-probability, LLCI-lower limit for 95% confidence interval; ULCI-upper limit for 95% confidence interval

Supplemental Table 2: Multiplicative interaction testing of age and gender: results of the multiple logistic regression model when the dependent variable was 'use of nutrition apps'

**Model**	**B**	**SE**	**P-value**	**LLCI**	**ULCI**
Constant	-32.4045	11.3285	0.0042	-54.6079	-10.2011
Age	1.5700	0.6488	0.0155	0.2984	2.8417
Gender	17.3007	6.0161	0.0040	5.5095	29.0920
Age x Gender	-0.9927	0.3524	0.0048	-1.6834	-0.3021
School program	0.3293	0.3169	0.2988	-0.2919	0.9505
GPA	0.0841	0.3311	0.7995	-0.5647	0.7330
Nutrition websites	1.3804	0.3376	0.0001	0.7188	2.0421
First internet use	-0.0788	0.0658	0.2308	-0.2077	0.0501

GPA-grade point average; B-unstandardized coefficient, se-standard error, p-probability, LLCI-lower limit for 95% confidence interval; ULCI-upper limit for 95% confidence interval
